# Outcomes after radical endoscopic resection of high risk T1 esophageal adenocarcinoma: an international multicenter retrospective cohort study

**DOI:** 10.1055/a-2538-9316

**Published:** 2025-04-28

**Authors:** Man Wai Chan, Rehan Haidry, Benjamin Norton, Massimiliano di Pietro, Andreas V. Hadjinicolaou, Maximilien Barret, Paul Doumbe Mandengue, Stefan Seewald, Raf Bisschops, Philippe Nafteux, Michael J. Bourke, Sunil Gupta, Pradeep Mundre, Arnaud Lemmers, Clémence Vuckovic, Oliver Pech, Philippe Leclercq, Emmanuel Coron, Sybren L. Meijer, Jacques J. G. H. M. Bergman, Roos E. Pouw

**Affiliations:** 1 522567 Gastroenterology and Hepatology, Amsterdam University Medical Centres, Amsterdam, Netherlands; 2 571143 Cancer Centre Amsterdam, Amsterdam, Netherlands; 3 571165 Amsterdam Gastroenterology Endocrinology Metabolism, Amsterdam, Netherlands; 4 4919 Division of Gastroenterology and Hepatology, University College London, London, United Kingdom of Great Britain and Northern Ireland; 5 591481 Digestive Diseases & Surgery Institute, Cleveland Clinic London, London, United Kingdom of Great Britain and Northern Ireland; 6 591481 Digestive Diseases and Surgery Institute, Cleveland Clinic London, London, United Kingdom of Great Britain and Northern Ireland; 7 2153 Division of Gastroenterology and Hepatology, Department of Medicine, Cambridge University Hospitals NHS Foundation Trust, Cambridge, United Kingdom of Great Britain and Northern Ireland; 8 2152 Early Cancer Institute, Department of Oncology, University of Cambridge, Cambridge, United Kingdom of Great Britain and Northern Ireland; 9 26935 Gastroenterology and Digestive Oncology, Hôpital Cochin, Paris, France; 10 Center for Gastroenterology, Hirslanden Clinic, Zurich, Switzerland; 11 60182 Gastroenterology and Hepatology, University Hospitals Leuven, Leuven, Belgium; 12 60182 Thoracic Surgery, University Hospitals Leuven, Leuven, Belgium; 13 8539 Gastroenterology and Hepatology, Westmead Hospital, Sydney, Australia; 14 1906 Gastroenterology, Bradford Teaching Hospitals NHS Foundation Trust, Bradford, United Kingdom of Great Britain and Northern Ireland; 15 70496 Gastroenterology, Hepatopancreatology and Digestive Oncology, Hopital Erasme, Brussels, Belgium; 16 155897 Gastroenterology, Regensburg Hospital of the Hospitaller Order of the Brothers of Saint John of God, Regensburg, Germany; 17 Gastroenterology, Clinique CHC Mont Légia, Liège, Belgium; 18 26922 Endoscopy and Gastroenterology, CHU Nantes, Nantes, France; 19 522567 Histopathology, Amsterdam University Medical Centres, Amsterdam, Netherlands

## Abstract

**Background:**

Post-endoscopic resection (ER) management of high risk T1 esophageal adenocarcinoma (EAC) is debated, with conflicting reports on lymph node metastasis (LNM). We aimed to assess outcomes following radical ER for high risk T1 EAC.

**Methods:**

We identified patients who underwent radical ER (tumor-negative deep margin) of high risk T1 EAC, followed by surgery or endoscopic surveillance, between 2008 and 2019 across 11 international centers.

**Results:**

106 patients (86 men; mean [SD] age, 70 [11] years) were included. Of these, 26 (age, 64 [11] years) underwent additional surgery, with residual T1 EAC found in five patients (19%) and LNM in two (8%). After a median [IQR] follow-up of 47 [32–79] months, 2/26 patients (8%) developed LNM/distant metastasis, with one EAC-related death (4%), one unrelated death (4%), and four patients lost to follow-up (15%). Of the 80 patients (age, 71 [9] years) who entered endoscopic surveillance, 5/80 (6%) developed LNM/distant metastasis, with four EAC-related deaths (5%) over 46 (IQR 25–59) months follow-up; there were 15 unrelated deaths (19%), and 10 patients lost to follow-up (13%). The overall rates (95%CI) were: LNM, 6% (2%–12%); LNM/distant metastasis, 7% (3%–13%); EAC-related mortality, 5% (2%–11%); overall mortality, 20% (95%CI 13–29).

**Conclusion:**

Our findings present low rates of LNM after radical ER of high risk T1 EAC, consistent with other endoscopy-focused studies. Post-surgical patients are still at risk for metastasis and disease-specific mortality. These results suggest that endoscopic surveillance is suitable for selected cases, but further prospective studies are needed to refine patient selection and confirm optimal outcomes.

## Abstract (de)

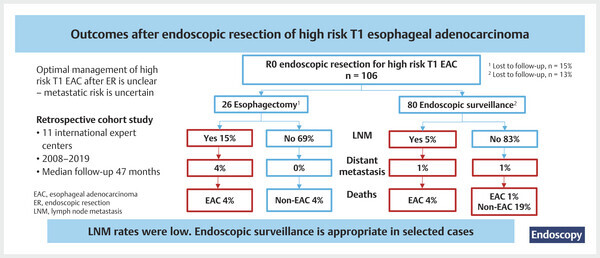

## Introduction

High risk T1 esophageal adenocarcinoma (EAC) is defined as: cancer invading the mucosa (T1a) with the presence of poor tumor differentiation or lymphovascular invasion (LVI); or cancer invading the submucosa (T1b) with or without these high risk features. The conventional approach for managing high risk T1N0M0 EAC has been surgical resection, involving esophagectomy and lymphadenectomy, to remove the cancer and any potential lymph node metastasis (LNM). However, esophagectomy carries considerable mortality (up to 6%) and morbidity rates (1.7%–49.5%), and may result in lifelong functional complaints, even in high volume centers 1 2 3 .

Recent advancements in endoscopic techniques have facilitated radical endoscopic resection (ER) of early esophageal cancers, even high risk T1 EAC, using methods like endoscopic submucosal dissection (ESD), which has become even more efficient when combined with traction techniques 4 5 . The optimal post-ER management for high risk T1 EAC is still debated owing to the uncertain but increased risk of LNM 6 . LNM rates of 0–46% for T1 EAC have prompted guidelines to recommend additional esophagectomy with lymph node resection 7 8 9 ; however, a small number of studies exploring endoscopic surveillance as an alternative post-ER approach have demonstrated its feasibility and safety for selected patients with favorable tumor characteristics (<500-µm invasion [sm1], no LVI, and well-to-moderately differentiated), particularly for those at high risk of surgical complications 4 10 11 . These endoscopy-focused publications report lower LNM rates for T1b EAC (0–16%), compared with earlier surgical series, although their small cohort sizes and mostly retrospective designs may have introduced bias. Further research is needed to clarify LNM risk and management outcomes.

This study aimed to assess the outcomes in a larger cohort of patients who, following radical ER for T1 EAC with at least one high risk feature, either underwent surgical resection or entered endoscopic surveillance.

## Methods

### Study design

This was a retrospective multicenter study involving 11 tertiary referral centers in Europe and Australia who were collaborating on large scale studies on early Barrett’s neoplasia management. The institutional review board (IRB) of the Amsterdam University Medical Centers declared that the study registry was not subject to the Medical Research Involving Human Subjects act, waiving the need for formal ethical review and patient consent. Each participating center’s IRB reviewed and approved the protocol.

### Study population

We identified patients who underwent ER for T1 EAC with at least one high risk feature between January 2008 and December 2019. Cases were mostly extracted from existing databases, although one center conducted a manual search. Notably, not all centers had initiated endoscopic mucosal resection (EMR) and ESD procedures by 2008.

Patients were included if they had a tumor-negative vertical (deep) resection margin (R0v). Tumor extension at the horizontal (lateral) margin was not considered an exclusion criterion, provided a radical ER had been performed. We categorized cases into the following three histologic risk groups.

- High risk T1a EAC ( **HR-T1a** ): mucosal EAC with poor-to-no differentiation (G3–4) and/or LVI
- Low risk T1b EAC ( **LR-T1b** ): submucosal EAC with superficial invasion (<500 µm; sm1), well-to-moderately differentiated (G1–2) and no LVI
- High risk T1b EAC ( **HR-T1b** ): submucosal EAC with deep invasion (≥500 µm; sm2–3), and/or G3–4, and/or LVI.

Exclusion criteria were: (i) tumor-positive (R1v) or inconclusive vertical resection margin; (ii) residual/metachronous tumor ineligible for endoscopic retreatment present at the first endoscopy following ER; (iii) baseline metastatic disease; (iv) prior EAC treatment; (v) use of chemo-/radiotherapy; (vi) no follow-up or management initiated; (vii) follow-up data unavailable.

This study did not include patients from the prospective PREFER study (NCT03222635), or Dutch patients from prior studies on this topic 12 13 . There is overlap with the prior cohorts in the studies of Graham et al. 14 (n = 8), and Benech et al. 15 and Doumbe-Mandengue et al *.* 16 (n = 10), although our cohort has a longer follow-up period.

### Endoscopic resection

**Fig. 1:**
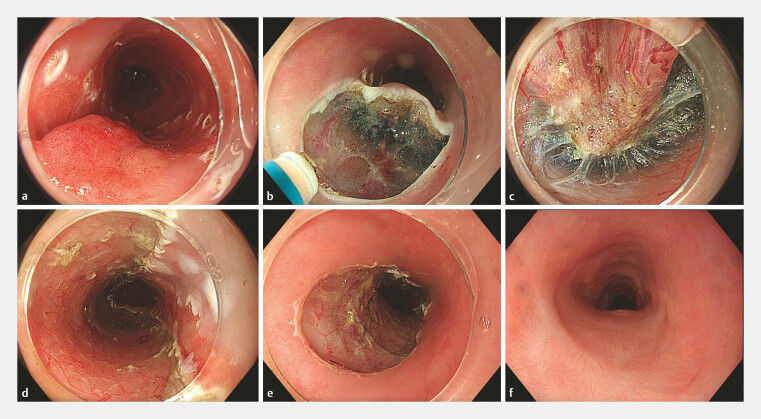
Endoscopic images of a high risk T1b lesion treated by endoscopic resection (ER) in an 81-year old patient with significant co-morbidity who entered endoscopic surveillance post-ER showing: **a** a Paris type 0-Is lesion of 25 mm in diameter within Barrett’s esophagus, delineated with electrocoagulation marks prior to endoscopic submucosal dissection (ESD); **b** the mucosal incision at the oral side of the lesion; **c** signs of deep submucosal invasion found during submucosal dissection; **d,e** the wound after radical ER of the lesion, which histologically was a radically resected (R0), poorly differentiated (G3) esophageal adenocarcinoma, invading over 2 mm into the submucosa (sm3), with signs of lymphovascular invasion; **f** the healed ESD scar with squamous mucosa at the restaging endoscopy 8 weeks after ESD.

ERs were conducted using cap- or band-assisted EMR techniques, or ESD by endoscopists with experience in managing Barrett’s neoplasia ( Fig. 1 ).

### Pathology assessment

ER specimens were assessed by experienced gastrointestinal pathologists adhering to the seventh edition of the International Union Against Cancer (UICC) TNM classification 17 . For T1a tumors, a distinction was made between those invading the lamina propria (m2) and the muscularis mucosae (m3). T1b tumors were categorized by depth of submucosal invasion: <500 µm (sm1) or ≥500 µm (sm2–3). The ER was considered radical if the vertical margin was tumor-free (R0v). In this study, we re-evaluated the endoscopy and pathology reports of all cases with surgically staged T1 disease initially marked as ER R0v.

### Staging examinations

During the inclusion period (2008–2019), staging and follow-up protocols for high risk T1 EAC varied and included endoscopies (with or without endoscopic ultrasound [EUS]) and computed tomography (CT) or positron emission tomography (PET)-CT scans based on physician preference.

### Post-ER management

#### Additional surgery

Surgical strategies, including minimally invasive and open thoracolaparoscopic esophagectomies, were chosen based on the tumor location and surgeon’s preference, with lymph node resection documented in most cases. Following surgery, a new TNM staging was determined.

#### Endoscopic surveillance

Conducted at the original ER center, surveillance endoscopies were scheduled at the treating physician’s discretion and included imaging (EUS, CT, and/or PET-CT) as needed.

### Study end points

The primary end point was the risk of LNM and distant metastasis during follow-up. Secondary end points were the rates of local recurrence requiring surgery in those under endoscopic surveillance, and disease-specific, other-cause, and overall mortality during follow-up.

### Data collection

Research fellows (M.D.) or nurses entered baseline and follow-up data on standardized forms in a joint online database (Castor EDC), with each institution maintaining a patient identification file. Missing data and illogical values were completed and corrected where possible. The database closed on 25 July 2023, with all authors reviewing and approving the final data.

### Statistical analysis

Statistical analysis was performed using IBM SPSS Statistics version 28.0.1.1 and R version 4.4.2. Descriptive statistics included mean (SD) for normally distributed variables, and median with interquartile range (IQR) for non-normal variables. Categorical variables are presented as counts with percentages. Exact 95%CIs for proportions were calculated using the exact binomial test in R to account for non-normal distributions. To compare subcohorts, in SPSS, the independent samples *t* test was used for continuous variables, and the chi-squared test for categorical variables, or Fisher’s exact test when expected cell counts were <5. All tests were two sided with a significance level of 0.05.

Follow-up duration was calculated from the initial ER to the last hospital contact, metastatic event, or death. Endoscopic follow-up was calculated from the initial ER to the last endoscopy. Kaplan–Meier was used for survival analysis, and the log-rank test was used to test for differences between subcohorts. The annual risk for recurrent disease was calculated by dividing the number of metastatic cases by the total follow-up time in years.

## Results

### Patient cohort

**Fig. 2:**
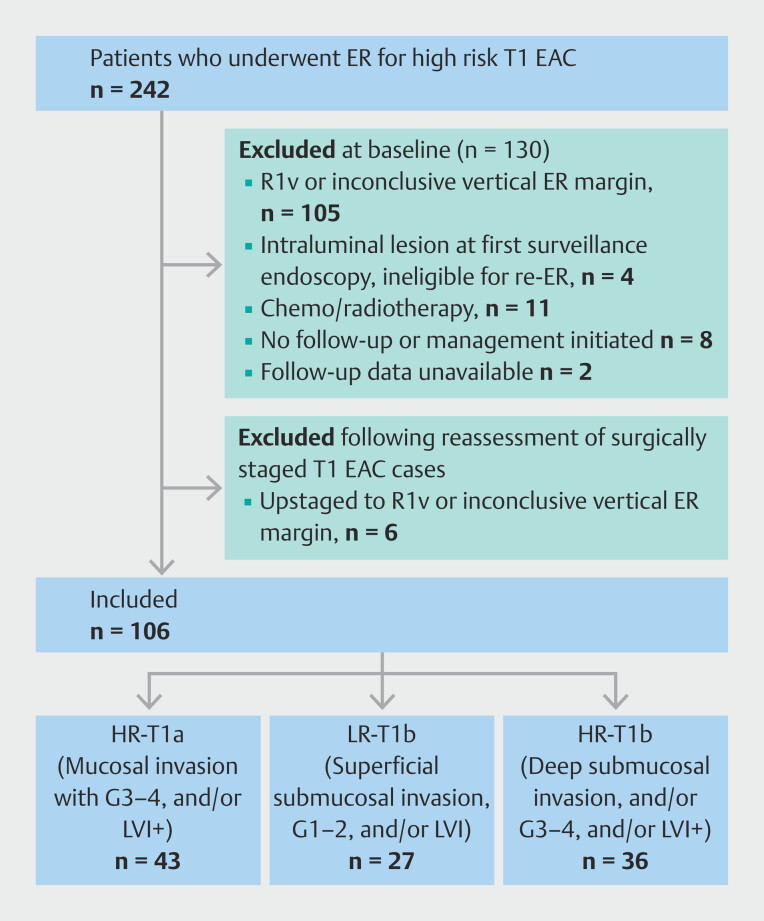
Flow of patient selection. EAC, esophageal adenocarcinoma; ER, endoscopic resection; FU, follow-up; G, tumor differentiation grade; HR-T1a, intramucosal EAC with poor/no differentiation and/or lymphovascular invasion; HR-T1b, submucosal EAC with ≥500-µm invasion, poor/no differentiation and/or lymphovascular invasion; LR-T1b, submucosal EAC with <500-µm invasion, well/moderately differentiated and no lymphovascular invasion; LVI, lymphovascular invasion; R1v, tumor-positive vertical ER margin.

Between January 2008 and December 2019, 242 patients underwent staging ER for high risk T1 EAC in Barrett’s esophagus, with 106 meeting the inclusion criteria (86 men; mean age at time of ER 70 years [SD 11]). Baseline endoscopic characteristics of the included patients are presented in Table 1 . Details of exclusions are shown in Fig. 2 . The excluded cases included six surgical cases that were initially categorized and included as ER R0v but, owing to evidence of (residual) T1 EAC in the surgical specimen, were reassessed, with there being clear arguments to register them as R1v or inconclusive, leading to their eventual exclusion ( **Table 1s** , see online-only Supplementary material).

**Table:** **Table 1** Baseline endoscopic characteristics of the 106 included patients.

	HR-T1a n = 43	LR-T1b n = 27	HR-T1b n = 36
Barrett’s length, median (IQR), cm			
Circumferential ^1^	1 (0–3)	3 (0–5)	1 (0–5)
Maximal ^2^	4 (2–7)	5 (1–8)	4 (1–6)
Tumor location ^3^			
Proximal esophagus (≤23 cm)	0	0	0
Mid-esophagus (24–32 cm)	4 (9%)	5 (19%)	5 (14%)
Distal esophagus (≥33 cm)	30 (70%)	18 (67%)	20 (56%)
Gastroesophageal junction	9 (21%)	4 (15%)	10 (28%)
Endoscopic resection techniques			
EMR	30 (70%)	18 (67%)	14 (39%)
#Multiband mucosectomy	26	15	14
#Endoscopic cap resection	2	1	0
#EMR technique unreported	2	2	0
ESD	13 (30%)	9 (33%)	22 (61%)
Tumor infiltration depth			
m2	4 (9%)	–	–
m3	33 (77%)	–	–
m, exact depth unknown	6 (14%)	–	–
sm1	–	27 (100%)	11 (31%)
sm2–3	–	0	25 (69%)
sm, exact depth unknown	–	0	0
Tumor differentiation grade ^4^			
G1	2 (5%)	5 (19%)	5 (14%)
G2	6 (15%)	22 (81%)	15 (42%)
G3	31 (72%)	0	15 (42%)
G4	3 (7%)	0	0
Lymphovascular tumor invasion			
Present	17 (40%)	–	9 (25%)
Absent	26 (61%)	27 (100%)	27 (75%)
Tumor diameter, mean (SD), mm ^5^	15 (± 8)	27 (± 23)	20 (± 11)
EMR, endoscopic mucosal resection; ESD, endoscopic submucosal dissection; HR-T1a, intramucosal EAC with poor/no differentiation and/or lymphovascular invasion; HR-T1b, submucosal EAC with ≥500-µm invasion, poor/no differentiation, and/or lymphovascular invasion; IQR, interquartile range; LR-T1b, submucosal EAC with <500-µm invasion, well/moderately differentiated, and no lymphovascular invasion. ^1^ Missing: HR-T1a, n = 2 (5%); HR-T1b, n = 2 (6%). ^2^ Missing: HR-T1a, n = 1 (2%); HR-T1b, n = 2 (6%). ^3^ Missing: HR-T1b, n = 1 (3%). ^4^ Missing: HR-T1a, n = 1 (2%); HR-T1b, n = 1 (3%). ^5^ Missing: HR-T1a, n = 7 (16%); LR-T1b, n = 11 (41%); HR-T1b, n = 16 (43%).			

Of the 106 patients, 26 (25%) underwent additional surgical resection following ER, while 80 (75%) entered endoscopic surveillance as they were deemed unfit for surgery (n = 31), or on the basis of the patient’s preference (n = 13) or local guidelines for low risk T1b (n = 20), with the reason unknown for 16 patients. Patients in the endoscopic surveillance group were older (mean [SD] age 71 [9] years) than those who underwent additional surgery (64 [11] years; *P* < 0.001). Endoscopic surveillance patients were also more frequently diagnosed with HR-T1a and LR-T1b ( *P* < 0.001), with no significant difference in their American Society of Anesthesiologists (ASA) classification ( *P* = 0.82).

### Surgical treatment after endoscopic resection

There were 26 patients (HR-T1a, n = 9; LR-T1b, n = 1; HR-T1b, n = 16) who underwent esophagectomy at a median (IQR) of 2 (1–3) months after ER. The esophagectomies were of the following types: minimally invasive thoracolaparoscopic, n = 14 (54%); open transthoracic, n = 6 (23%); open transhiatal, n = 2 (8%); and minimally invasive transhiatal, n = 1 (4%), with three unknown. Surgical morbidity was 65% (95%CI 49%–83%; n = 17), with infection being most common (n = 13; 50%), followed by anastomotic leakage (n = 3; 12%). The 30-day mortality was 0%.

In the esophagectomy specimens, invasive (residual) intraluminal cancer was found in five patients (19%; 95%CI 7%–39%): mucosal, n = 4; submucosal, n = 1. Nodal disease was found in two patients (8%; 95%CI 1%–25%), each with one positive lymph node, with a median (IQR) of 22 (17–29) nodes resected. Post-surgical staging showed T0N0M0 (n = 19), T1N0M0 (n = 5), and T0N1M0 (n = 2). Follow-up after surgery was a median (IQR) of 47 (32–79) months.

### Endoscopic surveillance after endoscopic resection

There were 80 patients (HR-T1a, n = 34; LR-T1b, n = 26; HR-T1b, n = 20) who entered endoscopic surveillance, with a median (IQR) of 7 (4–11) endoscopies performed over a median (IQR) of 41 (20–59) months. EUS, CT, and PET-CT were infrequently performed (median 0; IQR 0–1). The median (IQR) total follow-up was 46 (25–59) months.

### Metastatic disease

#### During follow-up

**Fig. 3:**
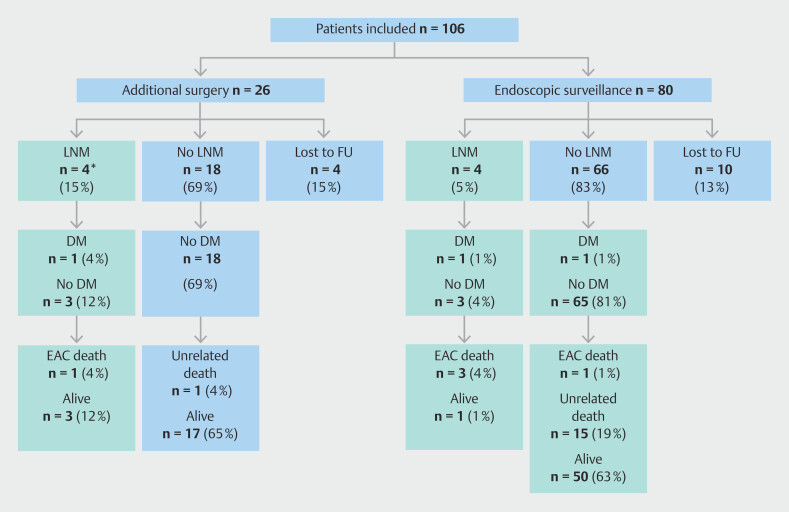
Flow diagram depicting the main follow-up outcomes. DM, distant metastasis; EAC, esophageal adenocarcinoma; FU, follow-up; LNM, lymph node metastasis. * Includes two cases diagnosed in the esophagectomy specimen.

There were 7/106 patients (7%, 95%CI 3%–13%) who developed LNM and/or distant metastasis during follow-up, diagnosed after a median (IQR) of 29 (12–38) months after the initial ER. The main patient outcomes are shown in Fig. 3 . In the surgical group, two patients showed LNM at 9 and 28 months post-esophagectomy, with one also having distant metastasis. In the endoscopic surveillance group, four patients developed LNM, with one simultaneously diagnosed with distant metastasis; the remaining patient developed distant metastasis after 38 months. The overall rate of LNM was 6% (95%CI 2%–12%), and of LNM and/or distant metastasis 7% (95%CI 3%–13%) over a median (IQR) of 47 (27–63) months of follow-up. The characteristics of these patients and the subsequent treatment of their metastatic disease are displayed in **Table 2s** .

#### Overall risk

Considering metastatic events following immediate surgery as well as during follow-up, metastatic rates were: for HR-T1a, 9% (4/43; 95%CI 3%–22%), with an annual risk during follow-up of 2.2% (95%CI 0.6%–5.6%); for LR-T1b, 4% (1/27; 95%CI 0.1%–19%), with an annual risk of 0.9% (95%CI 0.02%–4.9%); for HR-T1b, 11% (4/36; 95%CI 3%–26%), with an annual risk of 3.9% (95%CI 1.0%–9.6%) ( Table 2 ).

**Table:** **Table 2** Outcomes of patients during follow-up categorized by risk group.

Patients	All (n = 106)	HR-T1a (n = 43)	LR-T1b (n = 27)	HR-T1b (n = 36)
Duration of follow-up, median (IQR), months ^1^	47 (27–63)	52 (38–65)	50 (29–51)	36 (23–51)
Diagnosis of metastatic disease, n (%) [95%CI] ^2^	9 (8) [4–16]	4 (9) [3–22]	1 (4) [0.1–19]	4 (11) [3–26]
Annual risk of metastasis during follow-up (95%CI), %	2.2 (1.0–4.2)	2.2 (0.6–5.6)	0.9 (0.02–4.9)	3.9 (1.0–9.6)
Time to metastasis, median (IQR), months ^2^	29 (12–38)	12 (NA)	17 (NA)	33 (NA)
Disease-specific death during follow-up, n (%) [95%CI]	5 (5) [2–11]	3 (7) [1–19]	1 (4) [0.1–19]	1 (3) [0.1–15]
HR-T1a, intramucosal EAC with poor/no differentiation and/or lymphovascular invasion; HR-T1b, submucosal EAC with ≥500-µm invasion, poor/no differentiation, and/or lymphovascular invasion; IQR, interquartile range; NA, not applicable; LR-T1b, submucosal EAC with <500-µm invasion, well/moderately differentiated, and no lymphovascular invasion. ^1^ After initial endoscopic resection. ^2^ One case diagnosed in the esophagectomy specimen in the HR-T1a group and in the HR-T1b group.				

### Local intraluminal recurrence

Of the 80 patients in the endoscopic surveillance group, two (3%; 95%CI 0.3%–9%) required esophagectomy owing to intraluminal recurrence during follow-up exceeding re-ER limits, with post-surgical diagnoses of T1N0M0 and T3N0M0.

### Mortality

During follow-up, 5/106 patients died from EAC-related causes (one in the surgery group [4%; 95%CI 0.1%–20%] and four in the endoscopic surveillance group [5%; 95%CI 1%–12%]), resulting in a disease-specific mortality rate of 5% (95%CI 2%–11%).

Of the 106 patients, 16 died of non-EAC-related causes (one in the surgery group [4%; 95%CI 0.1%–20%] and 15 in the endoscopic surveillance group [19%; 95%CI 11%–29%]). Other-cause mortality was 15% (95%CI 9%–23%), with an overall mortality rate of 20% (95%CI 13%–29%).

**Fig. 4:**
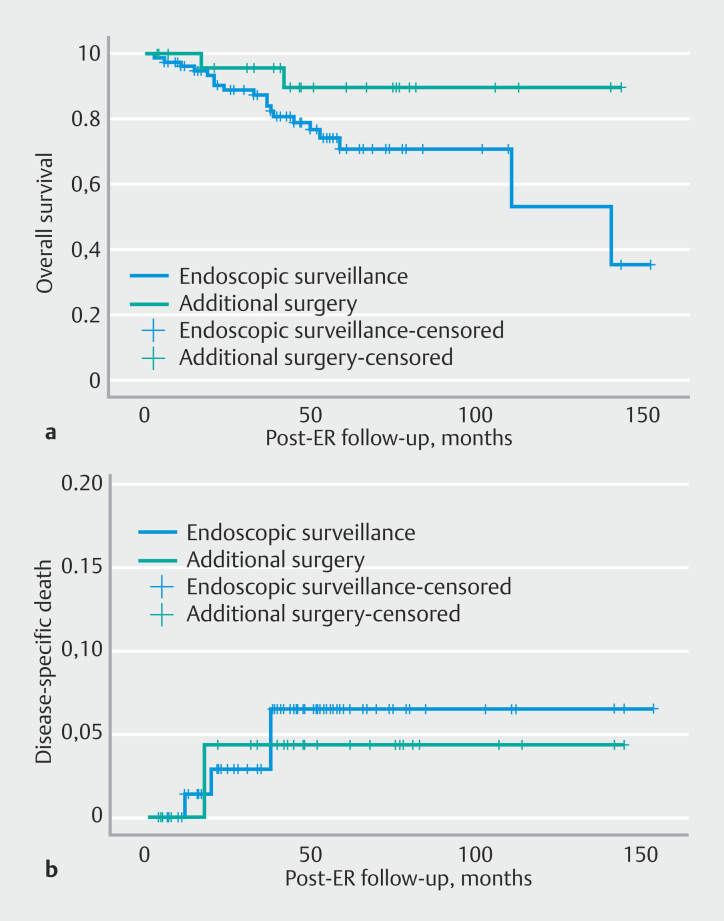
Kaplan–Meier survival curves for : **a** overall survival, showing no significant difference between the two subgroups (log-rank test; P = 0.07); **b** disease-specific deaths, showing no significant difference between the two subgroups (log-rank test, P = 0.80). ER, initial endoscopic resection procedure.

Kaplan–Meier analysis ( Fig. 4 ) suggested a potential trend toward better overall survival in the surgery group following ER (log-rank test, *P* = 0.07), considering all causes of death. Disease-specific mortality showed no significant difference between the two treatment subcohorts ( *P* = 0.80).

## Discussion

We conducted an international multicenter retrospective cohort study of 106 patients who underwent radical ER for high risk T1 EAC. In this cohort, EMR was performed more frequently (58%) than ESD (42%), with ESD becoming more common later in the study timeframe (2008–2019). The study encompassed outcomes from 26 patients who underwent additional surgery and 80 patients who entered endoscopic surveillance. Our cohort presents a considerable number of patients with relatively long follow-up periods. Within our study cohort, 29 patients had follow-up exceeding 5 years, and 10 more than 8 years, contributing to a cumulative 440 person-years of follow-up.

Our findings suggest low metastatic rates during follow-up. Notably, no significant difference in overall mortality rates was observed between the surgical and endoscopic surveillance groups, suggesting that additional surgery as post-ER management does not offer a survival advantage over conservative endoscopic management; however, the surgical group in our cohort was relatively small, and our results were not adjusted for age, pre-existing co-morbidities, or tumor stage.

These findings align with the previous literature, which shows that esophagectomy is not always a definitive curative approach for high risk T1 EAC, and metastatic disease can still occur. Westerterp et al. analyzed 120 T1 EAC patients undergoing esophagectomy (with nodal involvement in 19/120), without chemo-/radiotherapy, revealing 18 cases of recurrent disease over a median 44 months of follow-up, resulting in 10 deaths 18 . Molena et al. reported that among 23 T1b EAC patients undergoing esophagectomy (nodal involvement in 6/23 patients), with a median 37 months of follow-up, one patient died of systemic recurrence 19 . Schölvinck et al. found two recurrences among 25 patients with high risk T1b EAC who underwent esophagectomy (nodal involvement in 5/25 patients), over a 49-months follow-up period, both fatal 11 .

In addition, in our cohort, surgery did not appear to improve disease-specific mortality, aligning with the findings of Otaki et al. 20 . In their large multicenter study involving 141 T1b EAC patients, 68 underwent esophagectomy and 73 were managed endoscopically, with no correlation found between surgery and improved disease-free survival. Importantly, both studies lacked standardized follow-up protocols, limiting their conclusions. While in our cohort, overall survival seemingly favored the surgical group ( Fig. 4 ), the older age of the endoscopic surveillance group limits direct comparison, as does the absence of a standardized follow-up regimen.

The existing literature suggests that submucosal tumor invasion is likely associated with increased metastatic risks, with HR-T1b tumors carrying a higher risk than LR-T1b tumors. However, our data, albeit with limited case numbers, reveal low annual metastatic rates across all three risk groups, ranging from 0.8%–3.1% ( Table 2 ).

Regarding T1a EAC specifically, nonsurgical management has long been relatively consensual owing to the assumption of a very low to nonexistent risk of LNM (<1%) 10 11 21 22 ; however, studies specifically addressing HR-T1a are limited. Nieuwenhuis et al. reported a surprisingly high annual risk of LNM (6.9%, 95%CI 3%–15%) in their cohort of 25 HR-T1a patients undergoing ER (R0v) and surveyed for 35 (IQR 22–53) months 13 . Benech et al. included nine HR-T1a patients undergoing ER (R0v/R1v) who showed no metastatic disease during 35 (IQR 24–61) months of follow-up 15 .

In contrast, our larger HR-T1a subgroup (n = 43) with longer follow-up (median [IQR] 52 [37–65] months) exhibited an annual LNM risk of 2.2% (95%CI 0.6%–5.6%). Surprisingly, the metastatic rate during follow-up stood at 9% (95%CI 3%–22%), which exceeded our expectations. It is possible that the limited number of cases included and the lack of histopathology review might account for this, although this data, like the study by Nieuwenhuis et al., suggests that mucosal cancers with high risk features potentially carry a higher risk for metastasis than previously assumed.

Regarding metastatic risk of LR-T1b EAC, previous endoscopic cohort studies focusing on the long-term outcomes of this patient group have reported rates ranging between 0 and 2% 11 21 23 24 . Our present analysis echoes these findings, demonstrating a similarly low annual risk of 0.9% (95%CI 0.02%–4.9%) within this patient group. Although the observed metastatic rate during follow-up might appear relatively high at 4% (95%CI 0.1%–19%), this assessment is most likely owing to the small sample size (n = 27).

Concurrently, despite their limited cohort sizes and retrospective nature, an increasing number of endoscopy-focused studies report relatively low metastatic rates for HR-T1b, ranging from 0 to 16% 10 11 21 23 24 . Our findings regarding this patient subgroup align with these recent studies, displaying an annual risk of 3.9% (95%CI 1.0%–9.6%), which, although relatively low, exceeds the annual risks observed in our HR-T1a and LR-T1b subcohorts, as anticipated.

The metastatic rate of 11% (95%CI 3%–26%), based on a small sample size (n = 36), also falls within the anticipated range reported in the endoscopy-focused studies. Gotink et al. recently published a cohort study comprising 248 T1b EAC patients who underwent ER and/or surgery, assessing LNM presence in surgical resection specimens and during clinical follow-up 12 . In their cohort, one-third of patients experienced metastases within 5 years. Their scoring system, considering submucosal invasion depth, LVI, and tumor size, estimates a possible high metastatic risk of between 5.9% and 70.1% for T1b EAC. While we do advocate for a personalized risk model to advance personalized care, there are important limitations to their study design, such as the retrospective design covering mostly historical cases (1986–2016), handling of samples (lack of additional slide preparation in surgical specimens and no additional immunohistochemical staining). Moreover, the model relies predominantly on surgical data and may not be directly applicable to patients who undergo ER. Therefore, using these data for therapeutic decision-making is, in our opinion, not appropriate without external validation of the model in endoscopically treated patients.

The risk of metastatic disease in high risk T1 EAC has been reported to be as high as 46% in the literature 7 8 9 . Our rates, aligned with recent endoscopy-focused studies and involving extended follow-up durations, fall within the lower end of this spectrum, indicating low annual recurrence rates during follow-up. The discrepancy between surgical and endoscopy-focused studies may be attributed to differences in handling and processing surgical specimens versus ER specimens for pathologic diagnosis. Surgical specimens are cut at wider intervals, while ER specimens are fully embedded, raising the risk of underdiagnosis in surgical specimens. Additionally, advances in endoscopic imaging nowadays allow detection of more subtle high risk T1 lesions, which are then treated with ER. These subtle high risk T1 lesions may have different malignant potential compared with the more prominent high risk T1 lesions historically treated with surgery.

Furthermore, within our study, the extensive reassessment of 11 surgically staged T1 EAC cases initially registered as R0v at prior ER revealed the majority (6/11 cases) were mislabeled as R0v ER in the local registries. An in-depth reassessment of each case highlighted diverse reasons to reclassify as R1v or inconclusive, including pathology reports that were unable to confirm tumor-free vertical margins, endoscopy reports that indicated a metachronous lesion, and incomplete lifting during the ER, so preventing radical resection. These findings challenge previous retrospective studies that failed to thoroughly investigate such cases, thereby potentially missing misclassifications that might have contributed to higher reported rates of LNM.

This study has several key limitations. First, its retrospective design introduces potential selection and information biases, compromising the robustness of our findings. Second, there were no standardized baseline staging or follow-up protocols. Inconsistent use of EUS for LNM screening, coupled with infrequent imaging to assess for distant metastasis prior to initiating follow-up, could mean some patients already had baseline metastatic disease. Similarly, follow-up metastatic disease may have been undetected because of the low frequencies of follow-up imaging and EUS. Incorporating more rigorous follow-up visits with increased imaging examinations could potentially have identified metastatic disease at earlier, curable stages. Third, although the overall study population was substantial, the smaller subgroup sizes limit comprehensive comparative or predictive assessments for metastatic risk. Moreover, patients who underwent direct surgery without prior ER were not included, potentially skewing metastatic risk. Fourth, central pathology review was performed on only five selected cases ( **Table 1s** ). Finally, the retrospective nature of our study limited our ability to stratify mortality by pre-existing clinical factors.

The strengths of our study encompass its large multicenter cohort derived from 11 tertiary referral centers ( **Table 3s** ), making it, to our knowledge, center-wise the largest study on T1 EAC metastatic risk after ER. By including both surgical and endoscopic surveillance patients, it reflects real-world clinical practices, where nonsurgical candidates often receive endoscopic surveillance, enhancing the study's applicability. Additionally, the study uniquely focused on a well-defined cohort of high risk T1 EAC patients who underwent radical ER, excluding R1v and inconclusive resections after thorough re-examination of doubtful surgically staged T1 cases. The extensive median follow-up durations of 47 and 46 months strengthen the validity of our findings. While extended follow-up could potentially alter metastatic rates, this seems improbable given the median (IQR) post-ER time to diagnosis of metastatic disease was 29 (12–38) months. Prior studies also indicate metastases appear within 2 years post-ER 12 25 .

In summary, our study underscores the feasibility of a conservative organ-preserving endoscopic surveillance approach after radical ER for high risk T1 EAC as a valid alternative to surgical resection, in selected patients without baseline signs of residual cancer or metastatic disease. Our findings emphasize the need for re-evaluation of existing tumor risk factors to enhance risk stratification. The annual metastasis risks were low, but not negligible, across all three risk groups, which is consistent with recent endoscopy-focused studies, which have shown low metastatic incidences in high risk T1 EAC. Nonetheless, robust prospective data with standardized protocols and prolonged follow-up (PREFER study; NCT03222635) are requisite to ascertain the optimal management strategy and refine guidelines for the treatment of individuals with high risk T1 EAC.

## Supplementary Material

Supplementary Material Zusatzmaterial
