# Supplementary material for: Outcomes after radical endoscopic resection of high risk T1 esophageal adenocarcinoma: an international multicenter retrospective cohort study
Source: Endoscopy. 2025 Apr 28;57(7):699–708. doi: 10.1055/a-2538-9316 (PMC12224666; doi:10.1055/a-2538-9316)
Supplement: Supplementary file 1 — Supplementary Material [file 10-1055-a-2538-9316_25633800.pdf]

Outcomes after radical endoscopic resection of high risk T1 esophageal adenocarcinoma: an international multicenter retrospective cohort study

Man Wai Chan, Rehan Haidry, Benjamin Norton, Massimiliano di Pietro, Andreas Hadjinicolaou, Maximilien Barret, Paul Doumbe-Mandengue, Stefan Seewald, Raf Bisschops, Philippe Nafteux, Michael J. Bourke, Sunil Gupta, Pradeep Mundre, Arnaud Lemmers, Clémence Vuckovic, Oliver Pech, Philippe Leclercq, Emmanuel Coron, Sybren Meijer, Jacques Bergman, Roos Pouw

Contents

		Page
**Table 1s**	Details of the 11 patients with surgically staged T1 EAC	**2**
**Table 2s**	Patients that developed metastatic disease during follow-up	**3**
**Table 3s**	Study centers’ contribution to number of patients analyzed	**4**

Endoscopy | DOI 10.1055/a-2538-9316 | © The Author(s).

Table 1s. Following the exclusion of patients at baseline as per the criteria, 11 patients were identified with surgically staged T1 EAC (despite initially classified as radical ER, R0v). These cases underwent comprehensive reassessment through retrievable endoscopy, surgery, and pathology reports, of which five cases underwent additional pathological revision. Case-specific details are listed. The highlighted information reports an upstaged diagnosis of the deep endoscopic resection margin. ***ESD****: endoscopic submucosal dissection;* ***EMR****: endoscopic mucosal resection;* ***ER****: endoscopic resection;* ***G****: tumor differentiation grade,* ***LVI****: lympho-vascular invasion;* ***m****: mucosal infiltration;* ***R0v****: tumor-free vertical endoscopic resection margin;* ***R1v****: tumor-positive vertical endoscopic resection margin;* ***sm****: submucosal infiltration.*

Patients	Initial ER Diagnosis	Surgical TNM Stage	Acquired Information After Data Verification	ER Pathology After Pathology Revision	Patient Selection	Patient Outcome After Follow-up
1	T1sm1, G3, LVI+, R0v	T1bN0M0	Endoscopy report of ER procedure described incomplete resection (R1v). .	-	Excluded	Recurrence-free, alive.
2	T1sm1, G3, no LVI, R0v	T1aN0M0	Pathology report of ER specimen described inconclusive ER margin.	-	Excluded	Recurrence-free, alive.
3	T1sm1, G2, LVI+, R0v	T1bN1M0	Pathology report of ER specimen described inconclusive ER margin.	-	Excluded	Metastatic disease, EAC death.
4	T1sm1, G3, no LVI, R0v	T1bN0M0	Pathology report of ER specimen described radicality of <1mm to ER margin.	Diagnosis of tumor-positive ER margin (R1v).	Excluded	Recurrence-free until lost to follow-up.
5	T1sm3, G3, no LVI, R0v	T1aN0M0	Pathology report of ER specimen described radicality of <1mm to ER margin.	Diagnosis of tumor-positive ER margin (R1v).	Excluded	Recurrence-free, alive.
6	T1sm3, G2, no LVI, R0v	T1bN0M0	Pathology report of ER specimen described radicality of <1mm to ER margin.	Diagnosis of tumor-positive ER margin (R1v).	Excluded	Recurrence-free, alive.
7	T1m3, G3, LVI+, R0v	T1aN0M0	All reports described tumor-free ER margin (R0v).	Tumor-free ER margin confirmed (R0v).	Remained in study cohort	Recurrence-free, alive.
8	T1m3, G1, LVI+, R0v	T1aN0M0	Pathology reports irretrievable.	Tumor-free ER margin confirmed (R0v).	Remained in study cohort	Recurrence-free, non-EAC death.
9	T1sm1, G3, LVI+, R0v	T1aN0M0	ER pathology report described radicality of 0.2mm to ER margin.	-	Remained in study cohort	Recurrence-free, alive.
10	T1m3, G3, no LVI, R0v	T1aN0M0	ER pathology report described radicality of 0.3mm to ER margin.	-	Remained in study cohort	Recurrence-free, alive.
11	T1m2, G4, LVI+, R0v	T1aN0M0	ER pathology report described tumor-positivity in some ER fragments, but insufficient to alter R0v status.	-	Remained in study cohort	Metastatic disease, EAC death.

Endoscopy | DOI 10.1055/a-2538-9316 | © The Author(s).

Table 2s. Patients that developed metastatic disease during follow-up. ***ASA:*** *American Society of Anesthesiologists;* ***CRT:*** *chemoradiotherapy;* ***EAC:*** *esophageal adenocarcinoma;* ***ER:*** *(initial) endoscopic resection procedure;* ***FU****: follow-up;* ***G****: tumor differentiation grade,* ***HR-T1a****: intramucosal EAC with poor-no differentiation and/or lympho-vascular invasion;* ***HR-T1b****: submucosal EAC with ≥ 500 µm invasion, poor-no differentiation and/or lympho-vascular invasion;* ***LNM****: lymph node metastasis;* ***LR-T1b****: submucosal EAC with <500 µm invasion, well-moderate differentiation and no lympho-vascular invasion;* ***LVI****: lympho-vascular invasion;* ***m****: mucosal infiltration;* ***mo****; months;* ***NA****: not applicable;* ***sm****: submucosal infiltration.*

Case	Age at ER	ASA at ER	Baseline ER Diagnosis	Risk Group	Post-ER Policy	Type of Metastatic Disease	Follow-Up Till Diagnosis	Additional Therapy After Diagnosis	Outcome
1	62	II	T1sm1, G3, no LV	HR-T1b	Surgery	LNM (mediastinal) during FU	29 mo after ER/ 28 mo after surgery	CRT	Alive
2	56	III	T1m2, G4, LVI+	HR-T1a	Surgery	LNM and distant metastasis (bone) (simultaneously diagnosed) during FU	12 mo after ER/ 9 mo after surgery	Palliative care	EAC-related death (20 mo after ER)
3	69	III	T1sm2 G3, LVI+	HR-T1b	Endoscopic surveillance	LNM (truncal) during FU	39 mo after ER	CRT	Alive
4	66	II	T1sm1, G2, LVI+	HR-T1b	Endoscopic surveillance	LNM (truncal) during FU	33 mo after ER	Palliative care	EAC-related death (37 mo after ER)
5	78	II	T1m3, G3, no LVI	HR-T1a	Endoscopic surveillance	LNM (mediastinal) during FU	9 mo after ER	Palliative care	EAC-related death (11 mo after ER)
6	69	II	T1m3, G3, no LVI	HR-T1a	Endoscopic surveillance	Distant metastasis (liver) during FU	38 mo after ER	Palliative care	EAC-related death (38 mo after ER)
7	77	II	T1sm1, G2, no LVI	LR-T1b	Endoscopic surveillance	LNM and distant metastasis (simultaneously diagnosed, locations unknown) during FU	17 mo after ER	CRT	EAC-related death (19 mo after ER)

Endoscopy | DOI 10.1055/a-2538-9316 | © The Author(s).

Table 3s. Each study center's contribution in the number of patients included for the analysis.

Study center	Number of study patients included
1	12 (11%)
2	5 (5%)
3	5 (5%)
4	6 (6%)
5	21 (20%)
6	2 (2%)
7	1 (1%)
8	11 (10%)
9	25 (24%)
10	8 (8%)
11	10 (9%)
Total	106

Endoscopy | DOI 10.1055/a-2538-9316 | © The Author(s).
